# Physicochemical Mechanisms and Environmental Benefits of Using Basic Oxygen Furnace Slag for Sewage Sludge Stabilization

**DOI:** 10.1021/acsomega.5c13402

**Published:** 2026-04-14

**Authors:** Rosana G. Combarros, Esther González-Tolivia, Mario Díaz, Sergio Collado

**Affiliations:** a High School of Engineering and Technology, 247680 International University of la Rioja (UNIR), Av. De la Paz 137, Logroño 26006, Spain; b Department of Chemical and Environmental Engineering, 16763 University of Oviedo, Av Julián Clavería 8, Oviedo 33006, Spain

## Abstract

The increasing demand for inorganic alkalis in industrial processes, coupled with the environmental challenges of their production, has driven interest in sustainable alternatives. This study evaluates the feasibility of basic oxygen furnace (BOF) slag for sewage sludge stabilization by comparing its performance with commercial agents (NaOH and CaO) in batch hydrolysis experiments. The results reveal that BOF slag effectively increases the sludge pH to 12.4 at a dosage of 1.9 g/g of TSS. Although this dosage is higher than that required for NaOH (0.2 g/g) and CaO (1.0 g/g), BOF slag achieves comparable stabilization through a synergistic mechanochemical mechanism. Regarding heavy metals, BOF slag demonstrated superior immobilization capacity compared to caustic treatments; soluble lead (Pb) levels were maintained at 0.52 μg/g, significantly lower than the 9.2 μg/g release observed with NaOH. Pathogen reduction was notably effective, with *Salmonella* completely inactivated and coliforms reduced to <10 CFU/g using an optimal particle size of 0.6–1 mm. Furthermore, the economic assessment indicates that BOF slag reduces treatment costs by 15–30% compared with those associated with commercial alkalis. From an environmental perspective, the use of this byproduct results in a net carbon footprint reduction of 68–91% per ton of treated sludge, highlighting its potential as a cost-effective and low-carbon alternative for the circular economy in wastewater treatment.

## Graphical abstract

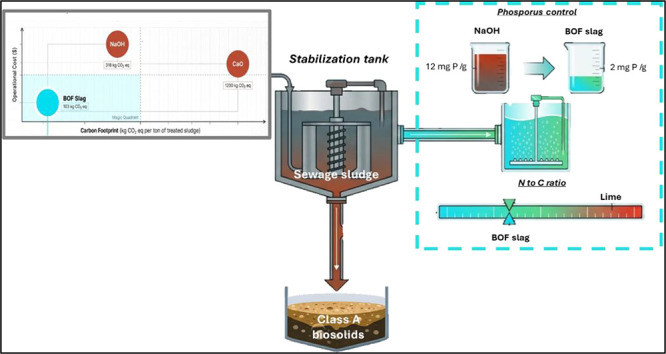

## 1 Introduction

It is beyond question that inorganic alkalis play a fundamental role across multiple sectors in the current chemical industry due to their huge versatility. Thus, caustic alkalis, such as sodium hydroxide (NaOH) and potassium hydroxide (KOH), are widely used in soap and detergent manufacturing, pulp and paper production, petroleum refining, and chemical synthesis. On the other hand, noncaustic alkalis, such as calcium or magnesium oxides and hydroxides, are essential for construction, environmental applications, metallurgy, and agriculture. This has led to a consistent, sustained rise in demand for these compounds, with projections indicating that the trend is set to continue in the coming years. To put this into perspective, global lime production reached an estimated 430 million metric tons in 2023, reflecting steady growth since 2010.^1^ Similarly, global production of caustic soda was approximately 100.6 million tonnes in 2022, with projections anticipating an increase to 112.6 million tonnes by 2028.^2^ In addition, the market size for magnesium oxide was valued at approximately 4363.2 million US dollars in 2023, and it is expected to grow significantly, reaching 8310.7 million US dollars by 2033.^3^

However, producing inorganic alkalis poses several challenges, including environmental impacts, operational inefficiencies, and safety concerns. One of the main disadvantages is that they are energy-intensive processes, which not only drive up production costs but also contribute substantially to the carbon footprint. This applies both to the Chlor-alkali process for the production of caustic alkalis and to the calcination of rocks for the production of noncaustic compounds.^4–7^ Thus, for every ton of caustic soda, 1.59 t CO_2_ eq is released, whereas total CO_2_ emissions per ton of quicklime are estimated to be around 1.2 t.^8,9^

At this point, it is also worth noting that the chemical industry simultaneously generates substantial quantities of alkaline waste and byproducts. For instance, industries such as steel production, alumina refining, and coal-fired power generation collectively produce approximately 2 billion tonnes of alkaline residues worldwide each year, representing a large and increasing global flux.^10^ These industrial wastes, which are usually stored in waste piles or landfills near production sites or storage facilities, entail a series of significant environmental hazards if not properly managed.^11^ Some of them are dust generation, heavy metal mobilization, alkaline leachate infiltration, or land degradation, among others.^10^

Both factors, namely, the increasing demand for alkalis as raw materials in industrial processes and the greater generation of alkaline wastes, have driven the search for new management strategies for these latter. Thus, in the interest of sustainability and advancing a circular economy, the research efforts in recent years to explore potential reuses for these residues have been highly commendable. The fields in which these efforts are being undertaken include construction materials, agricultural amendments, metal recovery, carbon sequestration, and other environmental applications.^10–13^

An excellent example of alkaline waste currently being explored for new valorization pathways is basic oxygen furnace (BOF) slag. This byproduct is generated during the steelmaking process when oxygen is blown through molten iron from a blast furnace to refine it into steel. BOF slag is primarily composed of oxides such as calcium, silicon, iron, magnesium, and aluminum, along with trace amounts of heavy metals.^14^ Beyond its alkaline nature and the potential presence of heavy metals, the management of BOF slag is also challenged by the substantial volumes produced. With global crude steel production reaching approximately 1.95 billion tons, the annual generation of steelmaking slags has exceeded 250 million tons,^15,16^ with BOF slag constituting the predominant fraction (72–80%)^14,17^ and generating around 150 kg per ton of steel produced.^14^ Regarding its comprehensive utilization status, while developed economies achieve high recycling rates, global utilization is often restricted to low-value applications; approximately 70% is directed toward construction materials,^15^ yet a substantial portion remains stockpiled due to technical limitations such as volumetric instability caused by free calcium and magnesium oxides.^16,17^ Other reuse fields include metal recovery, soil enrichment, and environmental remediation applications.^14,18–21^ With regard to this latter field, BOF slag has exhibited effectiveness in the treatment of industrial waters with high concentrations of heavy metals, sulfate, phosphate, arsenic, lead, and many other toxins.^18,22,23^ In parallel, the management of the substrate targeted in this study, sewage sludge, has evolved toward comprehensive resource recovery. Currently, sewage sludge is widely repurposed across various sectors, serving as a raw material for building and ceramic materials, a source of fuel, and an agricultural phosphate fertilizer. In the field of construction and engineering materials, recent studies have demonstrated significant progress in converting this waste into high-value products. For instance, Yu et al. successfully converted recycled sludge waste mixed with kaolin into high-value-added ceramics, achieving optimal performance with a 40 wt % sludge content.^24^ Further enhancing sustainability, the coutilization of sewage sludge with other industrial wastes has shown great potential; cost-effective ceramics with high mechanical strength were produced by combining kaolin, sewage sludge, and blast furnace slag, effectively reducing sintering temperatures.^25^ Moreover, advanced modification techniques, such as the use of anhydrous Na_2_CO_3_ to modify porous ceramics containing reclaimed blast furnace slag and urban sewage sludge, have significantly improved the physical and mechanical properties of eco-friendly porous materials.^26^ These advancements illustrate the versatility of sewage sludge as a renewable resource.

Nevertheless, available research on the potential applications of BOF slag in urban wastewater treatment plants remains highly limited. In fact, as far as we know, only two articles deal with this approach. Kim et al.^21^ explored the use of BOF slag to enhance alkaline sludge fermentation. Their findings demonstrated significant improvements in sludge hydrolysis, effective suppression of methanogenesis, and increased phosphate removal, highlighting the potential of BOF slag to stabilize pH levels and enhance the efficiency of waste treatment processes. González-Tolivia et al.^27^ also reported BOF slag-enhanced dewatering, solubilization, and pathogen reduction during secondary sewage sludge stabilization. Both studies underscore the significant potential of BOF slag as an effective material for use in the sludge treatment line of a conventional urban wastewater treatment plant. Nevertheless, there is a lack of studies comparing its performance to that of widely used commercial alkalis in sludge management, and existing studies do not provide in-depth analysis of the underlying mechanisms.

Building on previous findings, this study aims to compare the effectiveness of BOF slag in sewage sludge management with that of conventional commercial alkalizing agents. The insights gained will deepen our understanding of interactions between sludge and slag and evaluate the economic and environmental feasibility of integrating BOF slag as a sustainable alternative in conventional municipal wastewater treatment plants.

## 2 Materials and Methods

### 2.1 Materials

The BOF slag used in this study was sourced from a steel plant in Asturias, Spain. This material is highly heterogeneous, with primary components being CaO (48.9%), Fe_2_O_3_ (28.1%), and SiO_2_ (8.3%).^28^ For use, the slag was dried, milled, and sieved to a particle size of 710 μm. A more detailed characterization of the slag is available.^28^

A thickened secondary sewage sludge, processed via flotation, was collected from a municipal wastewater treatment plant in Asturias, Spain. After collection, it was stored at 4 °C for a maximum of 10 days before being utilized. The primary characteristics of this sludge are listed in Table 1.

**Table 1:** Properties of the Secondary Wet Sewage Sludge Utilized in This Study

parameter	mean values
pH	6.6 ± 0.1
total suspended solids (TSS_0_) (g/L)	34 ± 6
volatile suspended solids (VSS_0_) (g/L)	27 ± 4
total chemical oxygen demand (tCOD_0_) (g O_2_/L)	38 ± 5
soluble chemical oxygen demand (sCOD0) (g O_2_/L)	2.3 ± 0.8
soluble total organic carbon (sTOC_0_/ TSS_0_)	0.014 ± 0.009
soluble proteins (g/L)	0.31 ± 0.1
soluble carbohydrates (g/L)	0.06 ± 0.03
mercury (Hg) (mg Hg/kg dry matter)	0.11 ± 0.004
cadmium (Cd) (mg Cd/kg dry matter)	0.72 ± 0.002
chromium (Cr) (mg Cr/kg dry matter)	21 ± 0.5
lead (Pb) (mg Pb/kg dry matter)	33 ± 0.4
nickel (Ni) (mg Ni/kg dry matter)	43 ± 0.6
copper (Cu) (mg Cu/kg dry matter)	206 ± 3
zinc (Zn) (mg Zn/kg dry matter)	383 ± 8
aluminum (Al) (mg Al/kg dry matter)	8962 ± 161
iron (Fe) (mg Fe/kg dry matter)	16,647 ± 250
phosphorus (P) (mg P/kg dry matter)	17,334 ± 87

### 2.2 Experimental Setup

The experiments were conducted in 1 L jars equipped with a mechanical two-blade stirrer set to 250 rpm. Various agents (sand, NaOH, CaO, and BOF slag) were added individually and in pairwise combinations to sewage sludge. A control experiment in which no additives were added was also performed as a negative control. Throughout the experiments, samples were collected at regular intervals and stored at 4 °C until further analysis.

### 2.3 Analytical Methods

Samples were analyzed for pH, total chemical oxygen demand (tCOD), total suspended solids (TSS), and volatile suspended solids (VSS) following the procedures outlined in the Standard Methods.^29^

The alkalinizing capacity was determined by monitoring the pH in a 500 mL volume of sludge during gradual additions of the corresponding alkalinizing agent. The results were expressed as grams of agent per gram of total suspended solids.

For microbiological parameters, *Salmonella* sp. was quantified according to UNE-EN ISO 6579-1:2017/A1:2021 and total coliforms were determined using Chromocult agar.^30–32^

The liquid phase of the hydrolysate, separated from the solid fraction by centrifugation at 10,000*g* for 30 min, was used to analyze various parameters. Soluble COD (sCOD) was measured according to Standard Methods.^29^ Soluble proteins were determined using the Lowry method with bovine serum albumin (BSA) as the standard.^33^ Soluble carbohydrates were measured by the Dubois method using glucose as the standard, while reducing sugars were analyzed using the dinitrosalicylic acid method, also with glucose as the standard.^34,35^ Soluble total organic carbon (TOC) was determined using a TOC-V CSH analyzer (Shimadzu, Japan). To quantify the solubilization of heavy metals (Hg, Cd, Cr, Pb, Ni, Cu, Zn, Al, and Fe) during the experiments, an ICP-MS (Agilent 7700x, Agilent Technologies, California, USA) equipped with an integrated I-AS autosampler was employed. The collision/reaction cell operated with 4.3 mL/min of helium to eliminate interferences. The internal standards used were scandium (for Al, Cr, Fe, Ni, Cu, and Zn), rhodium (for Cd), and iridium (for Hg and Pb).

The same ICP-MS method was applied to determine the heavy metal content of the raw sludge. Prior to this analysis, a sludge sample was dried at 105 °C for 24 h and digested using the microwave acid digestion method. This involved adding 6 mL of HNO_3_ and 2 mL of a 30% H_2_O_2_ solution, followed by digestion in a microwave equipment (EthosOne, Milestone Systems, Denmark).

## 3 Results and Discussion

### 3.1 Alkalinising Properties

Prior to comparison of the effects of various alkaline agents on the physicochemical and biological properties of the sludge, their alkalinizing capacities were assessed. These capacities were quantified as the grams of alkaline agent required per gram of initial suspended solids in the sludge to reach a target pH (Figure 1). Notably, only treatments that successfully induced pH changes in the medium are presented in this section. Accordingly, data for control treatments, stirring, sand addition, and the combination of NaOH with sand are excluded from the results.

**Figure 1:**
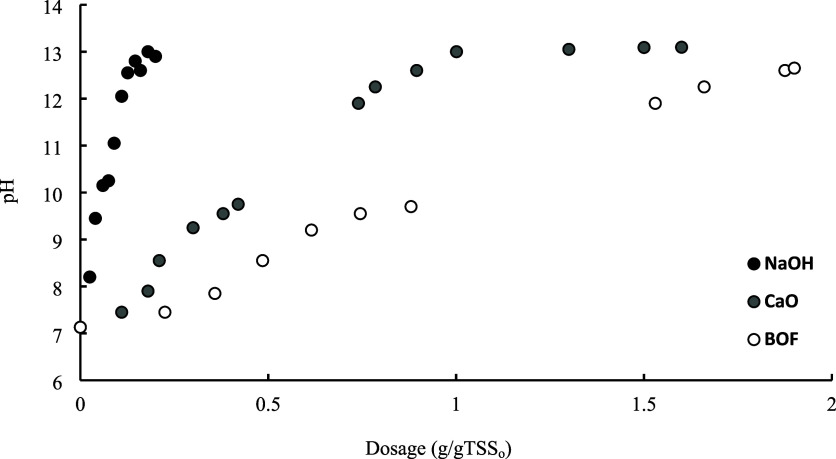
pH evolution of sewage sludge as a function of alkalizing agent dosage (room temperature, 500 mL of sludge, 250 rpm).

Unsurprisingly, Figure 1 corroborates that NaOH has the highest alkalinizing capacity as even a small dosage quickly raises the pH, reaching around 13.5 with just 0.2 g of NaOH per gram of TSS_0_. This rapid effect is attributed to NaOH’s high solubility in water. In contrast, CaO requires a much larger amount to achieve comparable pH increases. Thus, a CaO dosage of 1.0 g/gTSS_0_ is needed to achieve a pH of around 13.0 in the sludge. This is due to CaO's lower solubility in water, resulting in slower dissolution and a more gradual impact on the pH levels.

Focusing now on the BOF slag, it can be inferred from Figure 1 that its alkalinizing capacity is lower, requiring dosages of around 1.9 g of BOF per gram of TSS_0_ to reach final pH values close to 12.7. These findings differ slightly from those reported by González-Tolivia et al.,^27^ where stabilization was observed at a pH of around 12.9. This discrepancy can be attributed to the heterogeneity of the sludge, which varies in composition depending on the time of year. This variability is influenced by seasonal bacterial load and the nature of the waste discharged into municipal waters.^36–38^

Special mention should be made of the proportionality observed between the dosages of BOF slag and CaO. To achieve similar final pH values in the sludge, the gCaO/gTSS to gBOF/gTSS ratio ranges from 0.47 to 0.5. These ratios correspond to the 48% CaO content in BOF slag reported in the literature.^28^ This correlation helps explain the lower alkalinizing capacity of BOF slag compared with CaO when targeting the same final pH.

In order to better compare the different alkalinizing agents, all treatments from this point forward were carried out by dosing the necessary amounts of NaOH, CaO, or BOF slag to achieve a final sludge pH of approximately 13 (see Figure S1 in the Supporting Information). This pH value was selected as the sludge from wastewater treatment plants is considered to be stabilized (or inertized) when the pH is maintained above 12.^39^ This pH level is crucial for effectively reducing pathogen activity, which is key to ensuring safe handling and reuse of the sludge.

### 3.2 Solubilizing Properties

To explore the mechanisms occurring between sludge and BOF slag in more detail, Figure 2 illustrates the impact of various agents, both alkaline and nonalkaline, on the solubilization of the sludge after 1 or 2 h of treatment.

**Figure 2:**
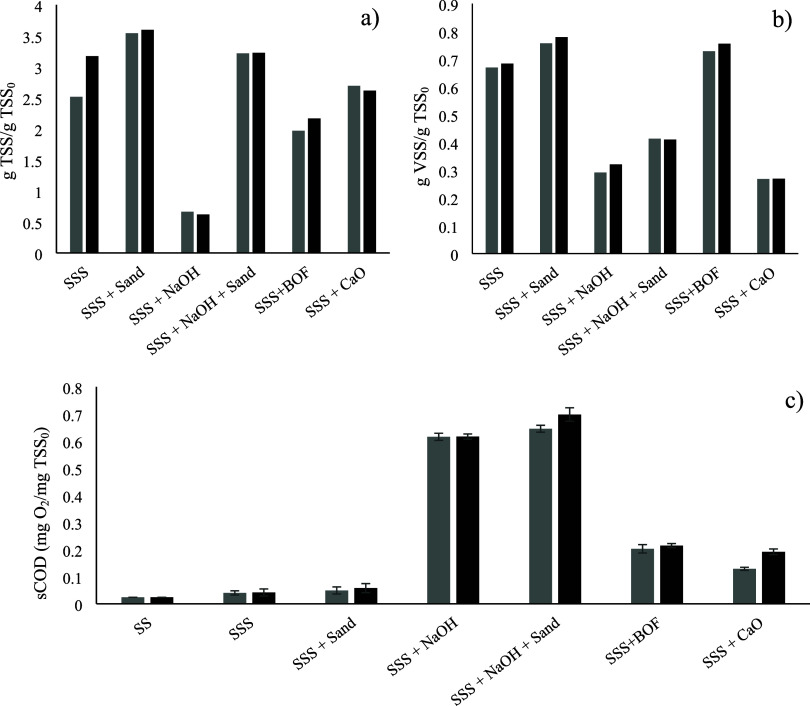
Evolutions of total (a) and volatile (b) suspended solids and soluble chemical oxygen demand (c) for the different treatments after 1 (gray bars) and 2 h (black bars). Sewage sludge (SS) and stirred sewage sludge (SSS). In all cases: room temperature, 500 mL sludge, and 250 rpm.

The results indicate that the solubilization degrees achieved in the experiments with sewage sludge (SS), agitated sewage sludge (SSS), and agitated sewage sludge with sand (SSS+Sand) were low and similar across all cases, regardless of the treatment time. These findings suggest that biological processes such as sludge autolysis can be ruled out during the experiments, which is not unexpected given the short duration of the tests. Additionally, the low solubilization observed in the agitated sludge and agitated sludge-sand experiments also discards the possibility of mechanical lysis at pH levels near neutrality, due to either shear forces generated by the agitator or shear effects caused by solid particles.

Focusing on experiments with different alkaline agents, Figure 2 clearly shows that NaOH induces the greatest sludge solubilization. The data strongly suggest that NaOH outperforms the other agents in promoting sludge breakdown, making it the most effective option for enhancing solubilization under these experimental conditions. For example, using NaOH as an alkalinizing agent to raise the pH of the sludge to 13 (at a dosage of 0.47 g NaOH/g TSS) resulted in a 27-fold increase in sCOD within less than an hour, with this elevated value remaining stable over extended contact times. These findings emphasize that the pH increase is the primary factor driving sludge disintegration.^40^ Furthermore, the complete dissolution of NaOH eliminates any potential abrasive effects in the experiment, confirming either an immediate or stable pH-induced effect. To isolate the contribution of mechanical abrasion, which is intrinsic to the granular BOF slag but absent in fully soluble NaOH, an experiment combining NaOH with inert sand was conducted. This setup aimed to simulate the physical collisions of slag particles under equivalent alkaline conditions. At this point, it is worth noting that the simultaneous addition of sand resulted in a further 13% increase in sludge disintegration compared to the experiment using NaOH alone. This suggests that shear effects, which were negligible at a near-neutral pH, become significant in alkaline sludge. This is because the sludge structure is already weakened by the alkaline conditions, making it more susceptible to mechanical abrasion from solid particles. In this regard, Şahinkaya et al.^40^ and Tian et al.^41^ demonstrated that combining NaOH treatment with ultrasonic disruption also produces a synergistic effect, significantly enhancing solubility values compared to alkali alone.

The use of CaO-based agents, such as quicklime and BOF slag, also led to notable solubilization, though the effect was significantly less pronounced than with NaOH. It is worth noting that neither quicklime nor BOF slag fully dissolved, suggesting additional potential for mechanical hydrolysis due to particle abrasion in the alkaline environment. However, the final sCOD was approximately three times lower than that achieved with NaOH alone.

This notable reduction in sludge solubilization with quicklime or BOF slag, compared with NaOH treatments, can be attributed to two intrinsic effects related to sewage sludge behavior in contact with Na^+^ or Ca^2+^ ions. First, the coagulation effects resulting from quicklime or BOF slag treatments are significant. Ca^2+^ ions act as a flocculant, capturing and retaining the organic matter present in the sludge. This reduction in soluble chemical oxygen demand (sCOD) has also been reported by other authors in industrial wastewaters treated with CaO for inertization.^42^ This flocculant-coagulant effect, also observed for BOF slag, is one of its key advantages over other alkalinizing agents for wastewater treatment, as the retention of organic matter leads to better outcomes for applications such as soil fertilization.^43^ This effect increases over time, explaining the slightly higher COD values observed after 2 h of treatment.^44^

Additionally, extracellular polymeric substances (EPS) are highly hygroscopic and act as a limiting factor for sludge dewaterability, as they disrupt the secondary structure of the proteins that make up EPS.^45^ Na^+^ interacts with N–H and C=O groups, reducing sludge's hygroscopicity and promoting the solubilization of organic matter, thereby increasing soluble COD (sCOD). It is important to note that this effect is less pronounced with Ca^2+^ ions.^46,47^

Focusing now on the alkalinizing agent under study, BOF slag, the results in Figure 2 show that it produces a level of solubilization comparable to, or even slightly higher than, that achieved with commercial quicklime. These results are consistent with expectations; as previously mentioned, BOF slag contains approximately 50% CaO.^27^ The slight improvement in soluble COD values observed in the earlier stages could be attributed to the higher abrasive action of BOF slag particles and/or its more heterogeneous composition, which may accelerate the sludge disintegration process compared to the use of CaO. In relation to this assumption, it is important to note that BOF slag contains significant amounts of MgO (5.03 ± 0.07%), MnO (1.60 ± 0.04%), K_2_O (0.39 ± 0.01%), and Na_2_O (0.125 ± 0.007%), which could explain the increased solubility, particularly due to the presence of these monovalent cations.^28^ However, the differences in sludge solubilization between quicklime and BOF slag were not substantial enough to confirm these hypotheses or to rule out the possibility of experimental error.

Once the capacity of each tested agent to disintegrate the sludge structure was determined, the next step was to determine whether they also caused preferential solubilization of some components over others. To answer this question, Figure 3 presents the composition of the resulting liquid phases, detailing the dissolved TOC, organic nitrogen, phosphorus, carbohydrates, and proteins for each tested agent.

**Figure 3:**
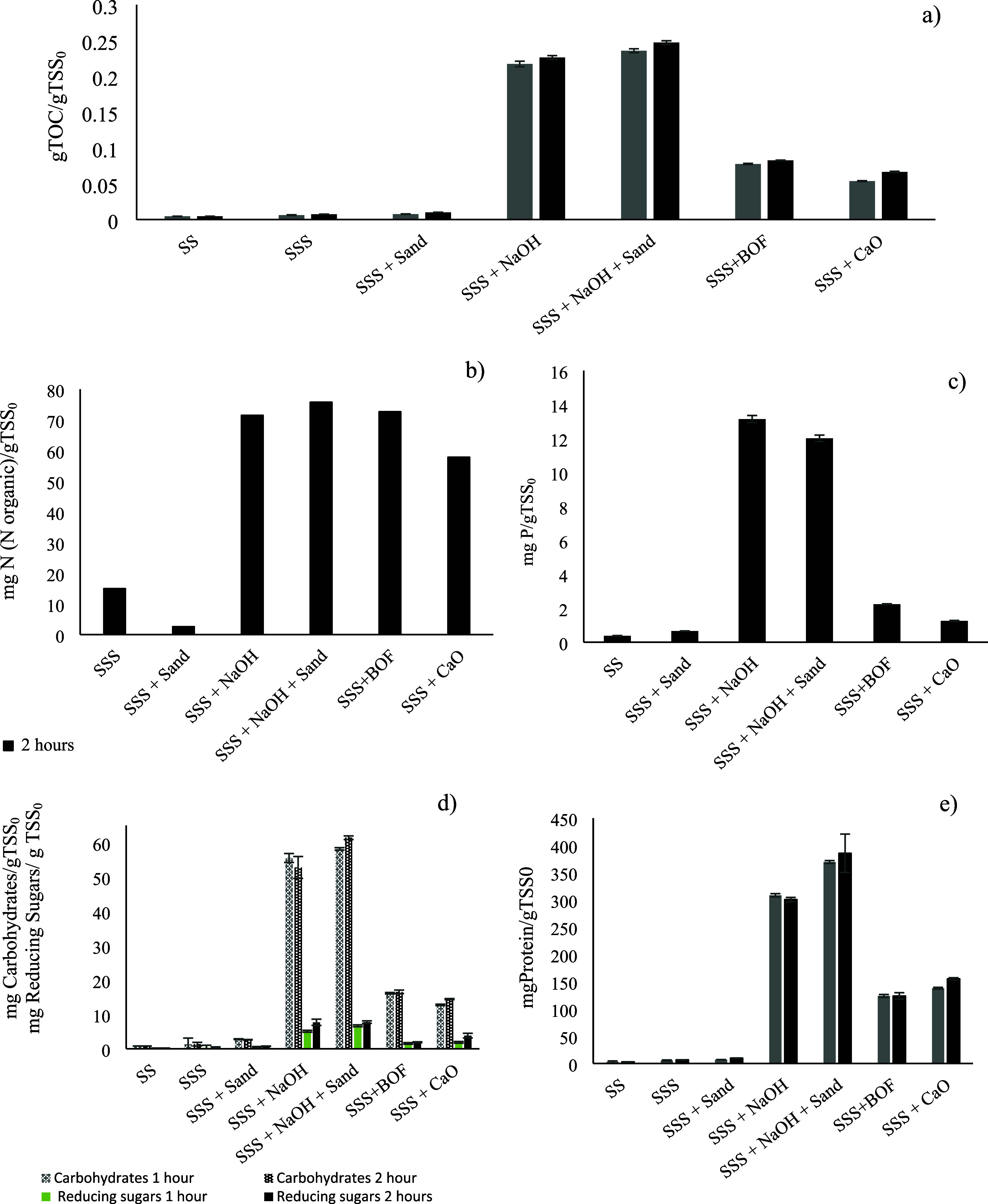
Evolutions of dissolved TOC (a), organic nitrogen (b), phosphorus (c), carbohydrates and reducing sugars (d), and proteins total (e) for the different treatments of alkaline hydrolysis after 1 (gray bars) and 2 h (black bars). Sewage sludge (SS), stirred sewage sludge (SSS). In all cases: room temperature, 500 mL sludge, and 250 rpm.

When examining soluble TOC, it follows a similar trend to the previously discussed soluble COD (see Figure 2), with higher solubilization with NaOH. If the TOC/sCOD ratio is evaluated, the results demonstrate that this parameter remains stable at approximately 0.18 across all treatments conducted without an alkalinizing agent. Notably, this value closely matches that observed in the initial sludge. However, in the presence of an alkalinizing agent, this parameter consistently assumes significantly higher values, approximately 0.38 for all agents tested. These results indicate that, in addition to enhancing sludge solubilization, alkalinizing agents also promote the release of compounds in a more reduced state. It has been shown that the inner fractions of microbial aggregates are inherently more susceptible to oxidation, with this being attributed to two mechanisms. First, the oxidative environment in the wastewater treatment plant exerts a screening effect on the more exposed EPS. Second, this outermost layer of EPS also acts as a physical barrier that mitigates free radical attack, thereby protecting the inner biopolymers and preserving their reduced state. Consequently, increased solubilization leads to the liberation of more internal, less oxidized floc components, which is reflected in a higher TOC/sCOD ratio.^48^

These findings are further supported by the concentrations of carbohydrates and proteins measured in the various hydrolysates, which serve as direct indicators of the EPS matrix disintegration. Unsurprisingly, greater solubilization was associated with increased amounts of these compounds in the final liquid phase. An analysis of the protein-to-carbohydrate ratio (g protein/g carbohydrate) revealed that it remained constant regardless of the alkaline agent′s cation. Specifically, the use of sodium (as NaOH) yielded ratios of 390 and 480 g of protein/g of carbohydrate without and with sand, respectively. In contrast, when CaO was used, either as lime or as BOF slag, the ratios decreased to 180 and 160 g of protein/g of carbohydrate, respectively. This higher protein solubility compared to carbohydrates is consistent with findings reported by several authors on EPS.^36,38,49^ These components form the structural framework of microbial aggregates and may represent as much as 80% of the total weight of the floc.^50^ Many studies have shown that EPS is the first component of floc to undergo solubilization. These substances are predominantly composed of proteins, with lesser contributions from humic acids and carbohydrates, thus corroborating the experimental results here observed.^49^

At this stage, it is noteworthy that the percentage of reducing sugars, relative to the total solubilized carbohydrates, consistently remained around 23 ± 3% for experiments without alkalinizing agents and 14 ± 5% for those with them. This suggests that no significant alkaline hydrolysis of carbohydrates into reducing sugars occurred after the agent used solubilization regardless of the alkalinizing.

The elemental compositions of the different hydrolysates were also analyzed. As expected, agents with higher sludge-solubilizing power produced hydrolysates with higher carbon, nitrogen, and phosphorus content. Nevertheless, it is worth noting that while CaO significantly enhanced nitrogen solubilization, its effect on phosphorus was limited. For reference, the concentration of soluble phosphorus in the initial sludge was 0.38 mg/g of dry matter. Treatment with NaOH increased this value substantially to 12 mg/g of dry matter, aligning with the greater sludge disintegration discussed in the previous section. In contrast, CaO resulted in significantly lower phosphorus solubilization with concentrations of 1.2 mg/g dry matter when using lime and 2.1 mg/g dry matter with BOF slag. This reduced phosphorus solubilization is attributed to the formation of low-solubility compounds, primarily hydroxyapatite (Ca_5_(PO_4_)_3_OH), due to the reaction between phosphate ions and calcium released by lime or BOF slag. On the other hand, the results show only slight differences among the alkalizing agents in terms of nitrogen solubilization. While the soluble nitrogen content in the final hydrolysate was slightly higher with NaOH compared to BOF slag or CaO, all treatments led to elevated levels, approximately 70 mg of nitrogen per gram of initial dry matter.^27^ These findings align with those reported by the literature, which stated that alkaline additives increase the total nitrogen concentration in the aqueous phase by promoting the decomposition of nitrogenous organic matter and the hydrolysis of nitriles.^51^

The elemental analysis also provides valuable insights into the potential management pathways for the hydrolysates. Liquid fractions separated from the sludge are typically returned to the water line of the treatment plant, specifically to the activated sludge biological treatment. These return streams have a significant impact on operating costs, particularly those associated with aeration during nitrification. In a conventional aerobic wastewater system, where oxygen is not limiting, the nutritional requirements for carbon (C), nitrogen (N), and phosphorus (P) have been reported to be approximately 100 (C):5 (N):1 (P).^39^ As shown in Table S1, which presents the amounts of carbon, nitrogen, and phosphorus obtained from the various hydrolysates, it can be concluded that carbon is the predominant element in all cases. Notably, the hydrolysates obtained using NaOH exhibit the lowest nitrogen/carbon ratio. Furthermore, the use of BOF slag resulted in a nitrogen-to-carbon ratio lower than that of lime, along with reduced solubilization. Given these factors, the management of hydrolysates derived from BOF slag is expected to be simpler and more cost-effective than that of hydrolysates obtained by using other alkalizing agents.

At this point, it is important to note that the impact of the alkaline pH in the liquid fractions obtained from sludge treatment with different alkalizing agents should also be accounted for in the subsequent processing of these streams within the wastewater plant. However, the dilution effect from mixing these fractions with the significantly larger inflow of wastewater entering the treatment plant helps to minimize potential negative impacts on activated sludge. Additionally, the supplied alkalinity could even have a positive effect on wastewater biological nitrification processes, as 7.14 mg of alkalinity is required for every milligram of ammonia converted to nitrate.

### 3.3 Stabilizing Properties

The sludge stabilization capacity of BOF slag, in comparison to other alkalizing agents, was also evaluated based on two key parameters: the immobilization of heavy metals and the reduction of pathogenic bacteria.

Regarding the first parameter, Figure 4 shows the concentrations of the main dissolved heavy metals in the sludge before and after treatment. The analysis initially focused on the major metals (Zn, Ni, Cu, Fe, and Al), with soluble concentrations measured in milligrams per gram of TSS. Fe and Al were the most abundant dissolved metals in all hydrolysates. Concentrations of 2.65 mg of Al/gTSS_0_ and 4.37 mg of Fe/gTSS_0_ were obtained using NaOH as the alkalinizing agent. However, upon employing BOF and CaO as alkalinizing agents, the concentrations were reduced to 0.048 mg of Al/gTSS_0_ and 0.20 mg of Fe/g of TSS_0_ and 0.0096 mg of Al/g of TSS_0_ and 0.046 mg of Fe/gTSS_0_, respectively.

**Figure 4:**
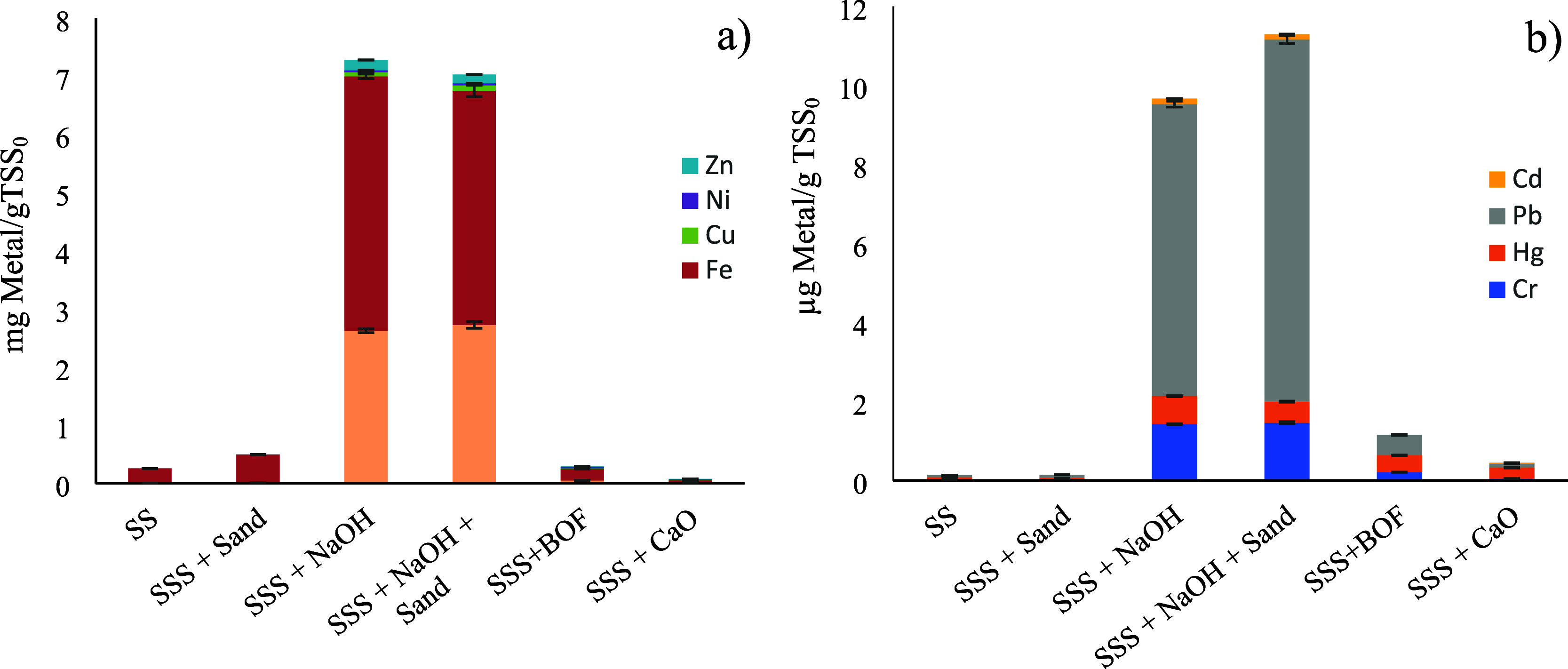
Heavy metal concentrations for the different treatments of alkaline hydrolysis: Zn, Ni, Cu, and Fe (a); Cd, Pb, Hg, and Cr (b). SS, sewage sludge; SSS, stirred sewage sludge. In all cases: room temperature, 500 mL sludge, and 250 rpm.

Both metals were also the primary components in the raw sludge (Table 1), consistent with these findings. As clearly shown in Figure 4, the concentration of dissolved metals in the untreated sludge is very low. This is entirely reasonable as the EPS matrix that forms the floc has not yet disintegrated. EPS are rich in negatively charged functional groups including carboxyl, hydroxyl, phosphoryl, and amide groups. These groups facilitate interactions with divalent metal cations, forming bridging structures that link microbial cells, EPS molecules, and other particles within the sludge matrix. Metals bind to these functional groups through mechanisms such as complexation and surface precipitation.^52^ When the EPS matrix was disintegrated, for instance, through the action of NaOH, metals were released back into the liquid phase. In contrast, when CaO is used, the sludge structure remains intact due to the presence of Ca^2+^ as a divalent ion, which prevents disintegration, as previously explained. These findings align with the experimental results, which showed that solubilization of heavy metals with NaOH was significantly higher, while the presence of CaO even reduced metal levels compared to untreated sludge. This latter effect can be attributed to the alkaline pH generated, which promotes the formation of metal hydroxides that precipitate and become less soluble. Focusing on BOF slag, the solubilization of these major heavy metals was also very low, as expected, given that it is composed primarily of CaO and, to a lesser extent, magnesium oxide. It is noteworthy that, when comparing BOF slag to quicklime, the concentration of dissolved metals, though still low, is higher in BOF slag. It is important to note that BOF slag contains a significant percentage of metal impurities, primarily iron and aluminum oxides, which likely explains this effect. In any case, the use of BOF slag did not result in a significant increase in dissolved heavy metal concentration compared to untreated sludge.

It is important to note that, despite the introduction of BOF slag, the final heavy metal concentrations in the stabilized sludge solids remain well below the regulatory threshold values for agricultural use established by European Directive 86/278/EEC. Furthermore, the low solubilization of metals observed in the BOF slag hydrolysates (Figure 4) represents a significant operational advantage; unlike caustic treatment, which releases metals into the liquid phase, BOF slag ensures that the return streams sent back to the biological treatment line do not carry toxic metal loads that could inhibit microbial activity. Regarding dissolved trace heavy metals (Cr, Hg, Pb, and Cd) present at concentrations of micrograms per gram of suspended solids but significantly more toxic than the previously mentioned metals, the results obtained are displayed in Figure 4. As observed, these metals exhibit similar behavior to that of the primary metals, showing maximum solubilization when NaOH is used and minimum solubilization when treated with quicklime. Again, the solubilization of metals with BOF slag was slightly higher than with lime, although it did not result in a significant increase compared to the levels found in untreated sludge or sludge treated only with sand. For instance, soluble Pb, the trace metal found in the highest proportion across all hydrolysates, increased from 0.056 μg of Pb/gTSS_0_ in the raw sludge to 0.52 μg of Pb/gTSS_0_ when treated with BOF slag. These values were notably lower than those achieved with the NaOH treatment, which yielded 9.2 μg/gTSS_0_. Therefore, it can be concluded that the use of BOF slag as an alkalinizing agent does not result in significant mobilization of heavy metals, unlike the noticeable effects observed when using NaOH.

Another fundamental aspect of sludge stabilization is the reduction of its pathogenic load. This reduction is essential to defining the treated sludge as biosafe and suitable for various applications without biological risks.^53,54^ In this regard, current European legislation *Regulation (EU) 2019/1009 of the European Parliament and of the Council of 5 June 2019* specifies that the density of *Escherichia coli* must not exceed 1000 colony-forming units per gram of total solids and that a 25 mL sample of sludge must be free of *Salmonella* to be deemed free of biological risk in organic compost.^55,56^

Therefore, in order to determine the effect of BOF slag on the biological stabilization of sludge in comparison to other agents, Figure 5 collects the coliform and *Salmonella* counts in the sludge after each treatment. In the absence of alkalizing agents, coliform counts for raw sludge ranged from 10^9^ to 10^10^ CFU/g TSS_0_, equating to approximately 4 × 10^11^ CFU/L sludge. As expected, mechanical treatments were insufficient for significantly reducing these pathogen populations in the sludge. This finding corroborates the hypothesis that pH is the primary determinant of sludge sanitization. As shown in Figure 5, the use of the commercially available alkalizing agents, NaOH and CaO, demonstrated to be a very effective method for the sanitization of sludge, with plate counts reduced to below 10^–4^ CFU/g TSS_0_ for both coliforms and *Salmonella*. These findings corroborate those of previous studies, which identified a pH of 12 as an effective means of significantly reducing pathogens, such as coliforms and *Salmonella*.^57^ BOF slag was similarly effective at removing *Salmonella* from sludge with efficiencies comparable to those of other alkalizing agents. Additionally, its use resulted in a significant reduction in the coliform bacterial count, reaching 6.7 CFU/g TSS_0_. Nevertheless, although its dosage resulted in a similar final pH value, this decrease in Coliform population was less pronounced than the complete reductions observed with the other alkalizing agents tested. Since the use of BOF slag as a sludge sanitizing agent is a novel approach, no references from other authors were found to explain this phenomenon. A plausible explanation for these results may well lie in both the heterogeneous composition and highly porous microstructure of the mineral fraction of BOF slag. These factors may serve as a niche for the adhesion of the sludge matrix microbiota, partially shielding them from exposure to Ca^2+^. To further investigate the mechanisms involved, an additional set of experiments was conducted, varying the dosage and/or particle size of the BOF slag added to the sludge. As anticipated, increasing the dosage of BOF slag while maintaining the selected granulometry resulted in a more significant reduction in coliform populations, particularly *E. coli*. Notably, increasing the amount of BOF slag added did not significantly change the resulting pH, which remained consistently around 13. Even more striking was the finding that the BOF slag particle size significantly influenced coliform counts. At the same dosage, almost complete elimination of coliforms was observed when particle sizes ranged from 0.6 to 1 mm. The use of larger or smaller particle sizes resulted in markedly lower biological stabilization of the sludge in terms of coliform reduction. However, the final pH and composition of the generated hydrolysates were very similar across the three particle sizes. Focusing specifically on the solubilization of heavy metals, the similar results strongly suggest that there are no compositional differences between particles based on their size. This suggests that the mechanism of coliform removal by BOF slag is not solely due to increased pH but also involves mechanical interactions between slag particles and flocs. In this process, both the number of particles and, more importantly, their size seem to play a crucial role. The results suggest that there may be a particle size distribution where the combination of collision frequency and associated momentum optimizes the impact of the slag particles on the flocs. This would enable greater disruption of the EPS matrix, facilitating direct contact between bacteria and the alkalizing agent and thereby leading to more effective bacterial removal. The reason coliform removal depended on particle size distribution, while *Salmonella* was completely eliminated in all experiments, was likely that *Salmonella* strains exhibited lower resistance to alkalizing agents than *E. coli*.^57–59^ Other studies have also concluded that for *E. coli*, a bacterium highly adaptable to adverse conditions, sodium-based alkalizing agents provide a more immediate bactericidal effect.^58–61^

**Figure 5:**
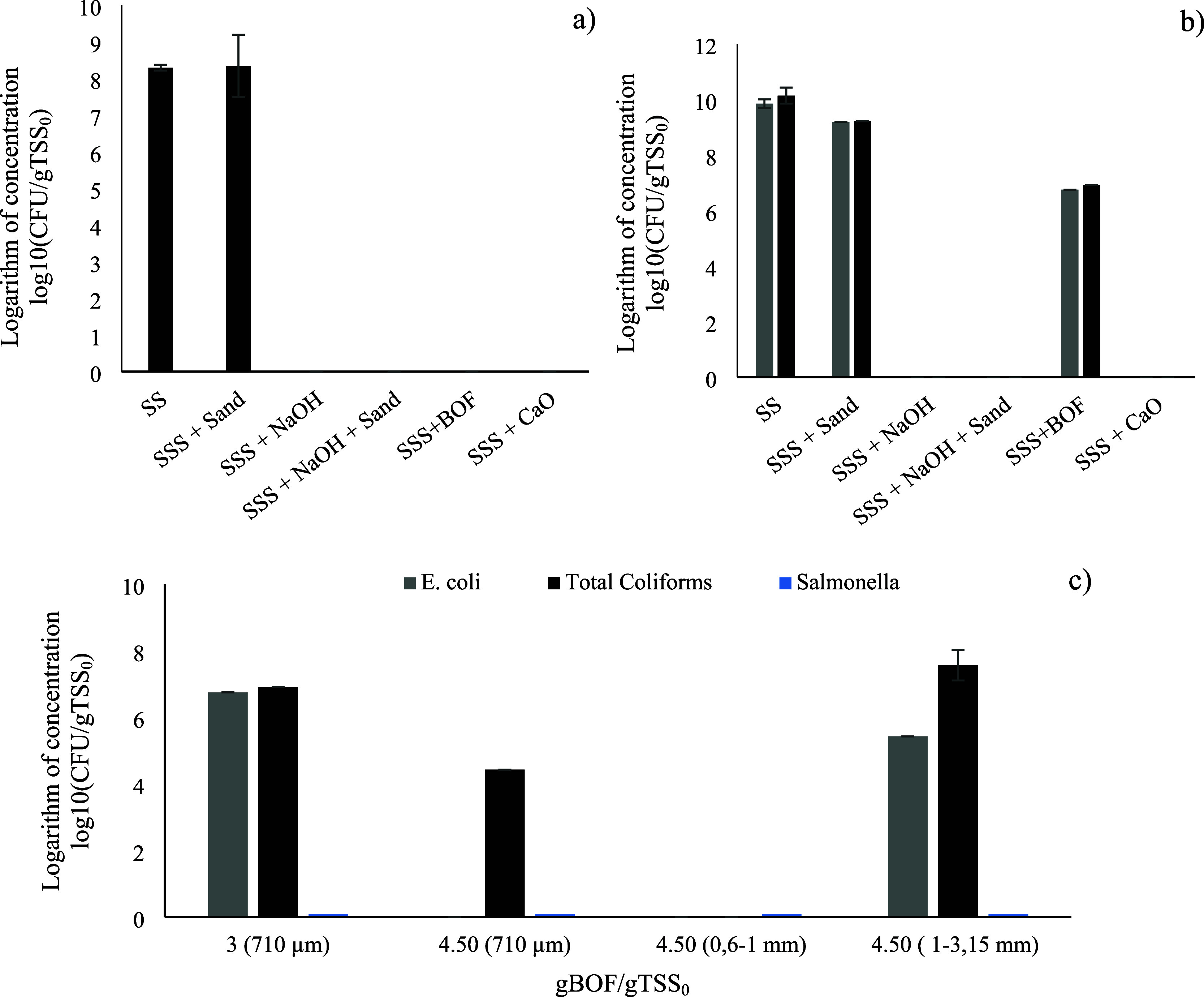
Presence of *Salmonella* sp. (a) *E. coli* (gray bars) and total coliforms (black bar) (b) for the different treatments of alkaline hydrolysis. (c) Presence of *Salmonella* sp. (blue bars), *E. coli* (gray bars), and total coliforms (black bars) for different concentration and particle size of BOF alkalisis. SS, sewage sludge; SSS, stirred sewage sludge. In all cases: room temperature, 500 mL sludge, and 250 rpm.

Therefore, in the context of sludge stabilization, BOF slag proved to be as effective as other alkalizing agents in reducing heavy metal mobilization and decreasing pathogenic bacterial populations. Although its efficiency in coliform removal was somewhat lower, this limitation was easily addressed by extending the contact time, optimizing the particle size distribution, or increasing the dosage, all without significantly affecting the associated costs.

### 3.4 Mechanisms

Based on the experimental findings, the interaction of BOF slag with sewage sludge appears to be governed by a combination of chemical and mechanical processes that facilitate sludge stabilization. This dual mechanism is supported by the differential performance of the inert abrasive control (NaOH+Sand), the particle-size-dependent pathogen reduction, and the protein-selective solubilization data.

Thus, the BOF slag, primarily composed of CaO, reacts with water from the sludge to produce calcium hydroxide (Ca­(OH)_2_), which increases the pH of the medium. This compound, despite its low solubility product, partially dissociates into Ca^2+^ and OH^–^ ions, reaching a maximum pH of 12.4. The release of other minor alkali compounds, such as magnesium and potassium oxides, may also contribute to the overall alkalinizing capacity and enhance the stabilization process. Nevertheless, this contribution can be deduced to be low due to the low concentration (potassium oxides) and/or low solubility products of these impurities (magnesium and iron oxides). Figure 6 illustrates the dual chemical mechanism at the microinterfacial level: While hydroxyl ions promote EPS hydrolysis and heavy metal precipitation, calcium ions induce a counter-active restructuring via cation bridging.

**Figure 6:**
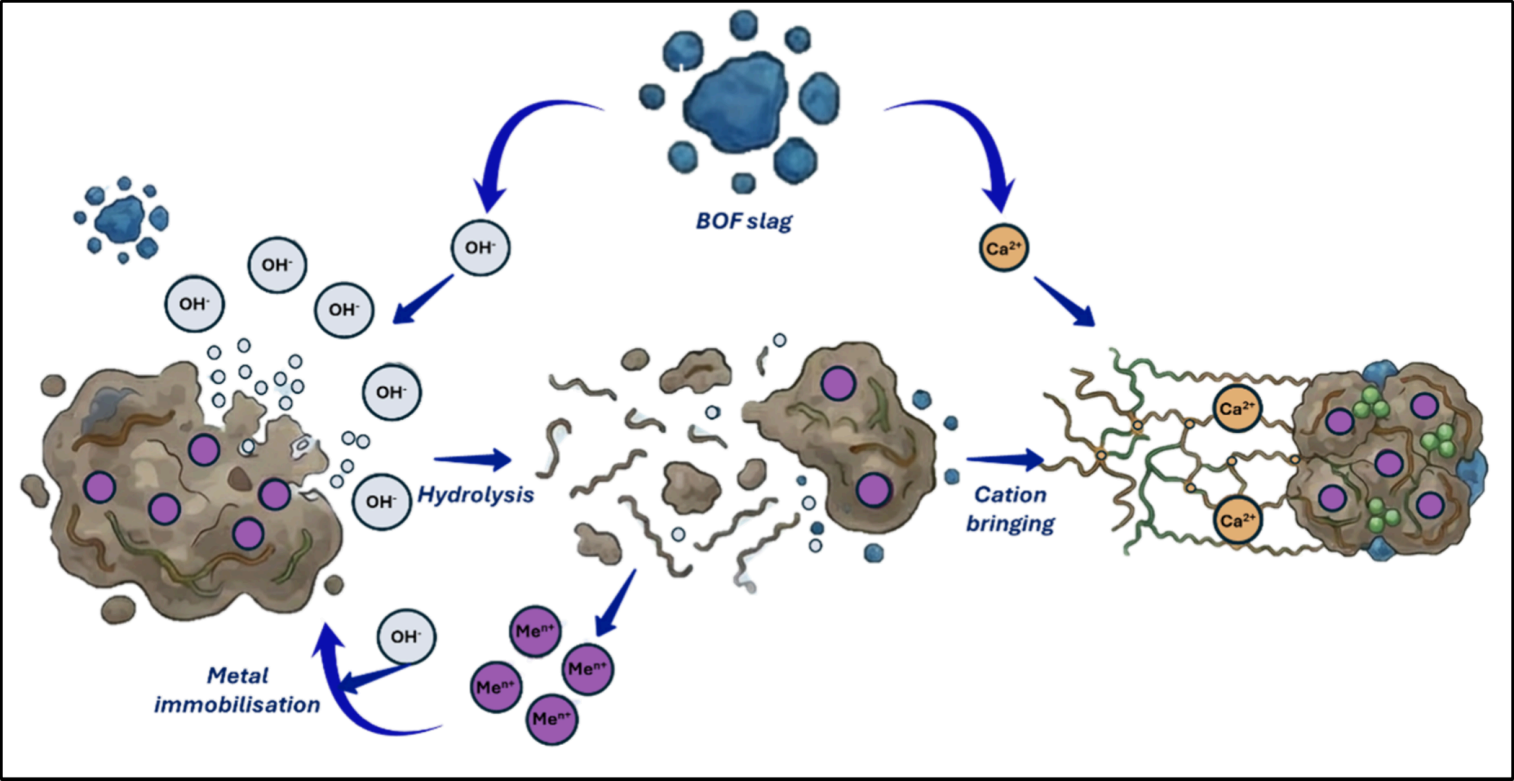
Role of the ions released by BOF slag particles in the stabilization of sewage sludge: hydrolysis mechanism and cation bringing.

The Ca^2+^ and OH^–^ ions released by the BOF slag played distinct roles in sludge stabilization (Figure 6). On one hand, the monovalent hydroxide ions disrupted the intermolecular forces that maintained the gel-like matrix of extracellular polymeric substances (EPS), which was essential for holding the floc structure together. Additionally, the high pH conditions created by BOF slag favored the precipitation of heavy metals as hydroxides. These insoluble hydroxides became immobilized within the sludge matrix, reducing their solubility and potential environmental risk. In contrast, Ca^2+^ exerts an effect on EPS that is opposite that of OH^–^ ions. As divalent cations, they form cross-links between the biopolymers constituting the gel-like matrix of the floc, enhancing flocculation and further trapping heavy metals.

The results also revealed a synergistic effect in floc destabilization driven by the combined action of hydroxide ions and the mechanical disruption caused by unsolubilized BOF slag particles. Figure 7 depicts the mechanochemical synergy. Under neutral conditions (a), flocs exhibit elastic resistance to mechanical shear. In purely chemical treatment (b), ionic bridging can shield bacteria. However, treatment (c) exploits the alkalin-induced structural weakening, allowing optimized slag particles to physically shatter the EPS matrix and expose pathogens to lethal pH levels. Thus, the alkalinization of the medium weakens the floc structure, making the shear effects generated by the slag particles more significant, an effect not observed under neutral pH conditions. Simultaneously, the mechanical disruption of sludge flocs exposes more bacterial cells to the alkalizing conditions, while the elevated pH ensures their inactivation. However, the exposure time of bacteria to the alkalinizing agent is mitigated by the flocculating effect of Ca^2+^ ions, as previously discussed. Thus, Ca^2+^ promotes the reaggregation of EPS around the bacteria, restoring its protective capacity against alkaline agents. These simultaneous phenomena of solubilization and reaggregation of the EPS matrix indicate that those microorganisms such as *E. coli*, which are more adaptable to adverse conditions, require additional mechanical disruption and prolonged exposure to achieve significant population reductions. This is not a critical issue if the bacteria are less resistant to pH, as is the case with microorganisms like *Salmonella*. Since BOF slag dosages exceeding 4.5 g/gSST_0_ did not significantly increase the resulting pH, optimizing mechanical disruption could be a decisive factor in inactivating more resistant microorganisms. The effectiveness of the BOF slag in removing coliforms is strongly influenced by the particle size distribution. Optimal particle sizes (∼0.6–1 mm) maximize the frequency and momentum of collisions with sludge flocs, leading to enhanced EPS disruption and bacterial removal. Larger particles may lack sufficient surface area for effective interaction, while smaller particles may not generate enough momentum for significant mechanical disruption.

**Figure 7:**
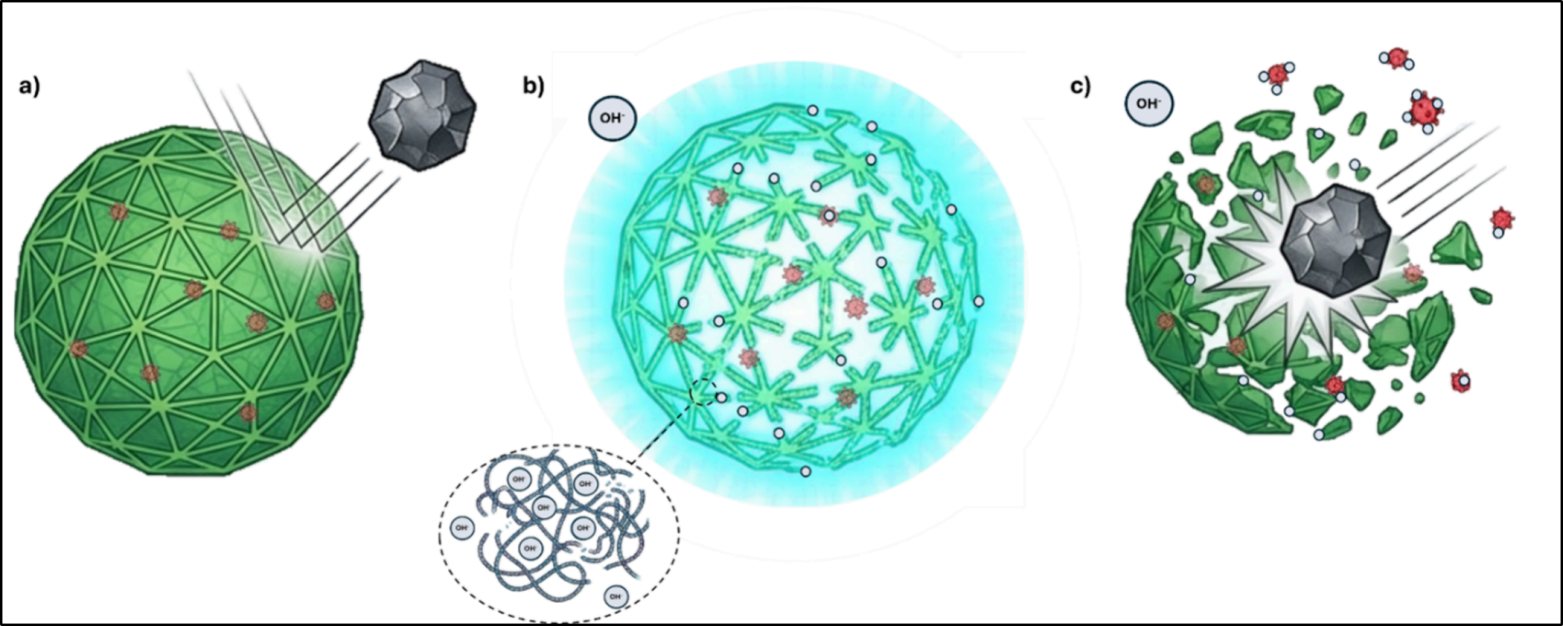
Synergistic effect in floc destabilization, driven by the combined action of hydroxide ions and the mechanical disruption caused by insolubilized BOF slag particles: (a) only mechanical effect, (b) alkaline solubilization, and (c) combined chemical and mechanical effects.

### 3.5 Economic and Environmental Implications

Finally, in order to evaluate the feasibility of BOF slag as a sustainable alternative to commercial alkalis, a comparative assessment of the economic viability and the carbon footprint was conducted, assuming a functional unit of 1 ton of dry sewage sludge (TSS) stabilized to a target pH of 12.7. Based on the experimental alkalinizing capacities determined in this study (Figure 1), the required dosages were established at 200 kg for NaOH (0.2 g/gTSS_o_), 1000 kg for CaO (1.0 g/gTSS_o_), and 1900 kg for BOF slag (1.9 g/gTSS_o_). While the experimental results indicated that BOF slag requires a nearly double dosage of CaO and 10 times that of NaOH, this mass inefficiency is offset by the disparate cost structures of the materials. Commercial alkalis, such as NaOH and quicklime, are manufactured commodities subject to volatile global market prices and energy-intensive production chains. In contrast, BOF slag is an industrial byproduct that often incurs negligible or negative acquisition costs due to the logistical burden of its disposal. This means that the primary costs associated with BOF slag utilization are restricted to transportation and mechanical pretreatment (drying and milling). Even when factoring in the necessary pretreatment costs identified by Ma et al.,^17^ specifically the energy consumption required for grinding and steam aging to ensure reactivity and volume stability (estimated at 100–180 kWh/t), the total expenditure for BOF slag treatment remains projected at approximately 15–30% of the cost associated with commercial alkalis. Consequently, the operational expenditure (OPEX) favors the use of slag, particularly in scenarios where transport distances from steelworks to wastewater treatment plants are minimized.

From an environmental perspective, replacing commercial alkalis with BOF slag represents a paradigm shift from a high-emission chemical production model to a lower-impact, energy-efficient one. BOF slag is considered to have a zero CO_2_ burden in its production phase since the associated environmental loads are allocated to the steel product. Nevertheless, as highlighted in recent reviews,^17^ the inherent hardness and density of BOF slag require intensive mechanical activation to enhance reactivity, necessitating a specific energy consumption for grinding of approximately 100 kWh/t. Furthermore, to mitigate the risk of volumetric instability caused by free CaO, stabilization protocols such as steam aging are recommended, which add an estimated 50–80 kWh/t to the process. Summing these requirements yields a total energy demand of roughly 180 kWh/t. By applying a standard industrial electricity emission factor of 0.30 kg CO_2_/kWh, the resulting carbon footprint for processed BOF slag is estimated at 54 kg CO_2_/t.

This value stands in stark contrast to the embodied carbon of commercial agents: The production of NaOH via the chlor-alkali process and CaO via calcination releases approximately 1590 and 1200 kg of CO_2_-equivalent per ton, respectively. When transposed to the operational context, the environmental advantage of slag becomes evident despite its higher dosage. To stabilize one ton of sludge (TSS), the required 0.2 tons of NaOH generate approximately 318 kg of CO_2_ eq. Similarly, the 1.0 ton of CaO required results in a footprint of 1200 kg of CO_2_ eq. In contrast, applying 1.9 tons of processed BOF slag yields a total footprint of only 103 kg of CO_2_ equiv per ton of treated sludge. Thus, the net carbon footprint is reduced by roughly 68% compared to NaOH and over 91% compared to CaO, confirming the superior sustainability of the slag-based treatment.

Finally, the integration of BOF slag supports advanced circular economy strategies by delivering both operational and environmental benefits. Hydrolysate analysis revealed that BOF slag treatment yields a lower nitrogen-to-carbon ratio in the supernatant lower than that of lime-based treatments. When these streams are returned to the biological treatment line, the reduced oxygen demand for nitrification translates into a lower aeration energy consumption at the wastewater treatment plant. In parallel, recent advances in slag valorization describe the potential of applying magnetic separation prior to alkaline use to recover valuable metallic fractions such as iron and vanadium.^17^ Incorporating this step could help offset grinding costs while further limiting heavy metal accumulation in the sludge, ensuring that the stabilized material remains within safe agricultural limits. Overall, BOF slag shifts sludge management away from the linear consumption of costly reagents toward a more resource-efficient scheme that valorizes industrial byproducts and contributes to reducing the carbon footprint of the water sector.

With regard to downstream management, the addition of BOF slag also influences the most suitable disposal or reuse routes for the treated sludge. Although the required dose (1.9 g/g of TSS) increases the total solid mass, the resulting composite exhibits clear advantages for land application. Its high calcium and iron content, together with effective heavy metal immobilization and pathogen inactivation, yields a safe, nutrient-rich amendment capable of correcting soil acidity. From an operational perspective, the granular slag particles act as a structural skeleton, improving sludge dewaterability and producing a drier filter cake, thereby limiting the practical impact of the increased mass. Alternatively, the inorganic framework enhances the material′s suitability for construction-related applications (e.g., brick manufacturing), in line with resource recovery strategies. In addition, the residual, unreacted silicates retain the capacity to mineralize carbon upon exposure to atmospheric CO_2_, providing a passive carbon sequestration pathway during final disposal or reuse.

## 4 Conclusions

This study shows that basic oxygen furnace (BOF) slag is a technically and economically viable alternative to conventional alkalizing agents such as NaOH and CaO for sewage sludge stabilization. Although higher dosages are required, BOF slag reliably achieves pH conditions sufficient for pathogen inactivation and heavy metal immobilization, keeping metal concentrations within regulatory limits for agricultural use. Despite its lower solubility relative to NaOH, the release of OH^–^ and Ca^2+^ plays complementary roles: Hydroxide ions disrupt the EPS matrix and promote organic matter and metal solubilization, while calcium ions enhance flocculation and metal retention, with the balance between these effects determining the overall stabilization efficiency.

In parallel, the abrasive action of undissolved slag particles mechanically disrupts sludge flocs, increasing the level of bacterial exposure to the alkaline environment. This combined mechanochemical mechanism enhances inactivation of pH-sensitive pathogens such as *Salmonella*, although Ca^2+^-driven reaggregation may partially shield more resistant bacteria such as *E. coli*. Consequently, optimizing dosage, contact time, and especially particle size is essential: Slag fractions in the range of 0.6–1 mm maximize collision frequency and momentum, strengthening both pathogen removal and metal immobilization. While BOF slag exhibits slightly lower coliform removal than commercial alkalis, this limitation can be mitigated through such operational optimization while retaining the advantages associated with its significantly lower carbon footprint and reduced operating costs.

Beyond primary stabilization, BOF slag also influences downstream sludge management and valorization routes. The stabilized sludge, enriched in calcium and iron, with immobilized heavy metals and inactivated pathogens, can act as a safe, nutrient-rich soil amendment capable of correcting acidity or as a component in construction materials, while the granular slag framework improves dewaterability and yields a drier filter cake that partly offsets the increase in total solids. Residual unreacted silicates retain the capacity to mineralize carbon for mineral carbonation upon exposure to atmospheric CO_2_, providing passive carbon sequestration during final disposal or reuse. In combination with emerging strategies such as magnetic separation to recover valuable metals from slag prior to its use, these features position BOF slag as a key element in the transition from linear, reagent-intensive sludge management toward a circular, resource-efficient approach in the water sector.

## Supplementary Material



## Notes

pH evolution during alkaline hydrolysis experiments (Figure S1); concentrations of C, N, and P in hydrolysates after different treatments (Table S1) (PDF)
