# Supplementary material for: Physicochemical Mechanisms and Environmental Benefits of Using Basic Oxygen Furnace Slag for Sewage Sludge Stabilization
Source: ACS Omega. 2026 Apr 14;11(16):24436–48. doi: 10.1021/acsomega.5c13402 (PMC13129847; doi:10.1021/acsomega.5c13402)
Supplement: Supplementary file 1 [file ao5c13402_si_001.pdf]

Supplementary material for

Physicochemical Mechanisms and Environmental Benefits of Using Basic Oxygen Furnace Slag for Sewage Sludge Stabilization.

Rosana G. Combarros[a], Esther González-Tolivia[b], Mario Díaz[b], Sergio Collado[b*]

[a] High School of Engineering and Technology, International University of la Rioja (UNIR) Av. De la Paz 137, 26006, Logroño, Spain

[b] Department of Chemical and Environmental Engineering, University of Oviedo, Av Juián Clavería 8 33006, Oviedo, Spain

[*] Corresponding author: Sergio Collado (colladosergio@uniovi.es).
Department of Chemical and Environmental Engineering, University of Oviedo, c/Julián Clavería 8, 33006 Oviedo, Asturias, Spain

Index	***Page***
S1.- Physicochemical Characterization of Basic Oxygen Furnace (BOF) Slag	3
S2.- pH evolution of the different alkalising agents	5
S3.- Soluble concentration of C, N and P after different treatments of alkaline hydrolysis.	6

S1.- Physicochemical Characterization of Basic Oxygen Furnace (BOF) Slag

To support the mechanistic discussion regarding the chemical alkalization and mechanical disruption of sludge flocs presented in the main manuscript, the physicochemical properties of the BOF slag used in this study are detailed below. This material originates from the same batch extensively characterized in previous studies by the authors [1, 2].

S1.1.- Chemical Composition (XRF)

The elemental composition of the BOF slag was determined by X-Ray Fluorescence (XRF). The material is primarily composed of calcium and iron oxides, which are responsible for the alkalizing capacity and magnetic properties, respectively.

Table S1. Chemical composition of the BOF slag (major oxides). Data adapted from González-Tolivia et al. (2022) and Matthaiou et al. (2019).

Component	Content (wt%)
**CaO**	48.9 ± 0.5
**Fe_2O_3**	28.1 ± 0.3
**SiO_2**	8.3 ± 0.2
**MgO**	5.03 ± 0.07
**MnO**	1.60 ± 0.04
**K_2O**	0.39 ± 0.01
**Na_2O**	0.125 ± 0.007

S1.2.- Mineralogical characterization (XRD)

The crystalline phases present in the slag were identified via X-Ray Diffraction (XRD). The detailed diffraction pattern for the raw slag (Sample S1) can be found in Figure 1a of Matthaiou et al. (2019).

The analysis reveals a complex mineralogical matrix containing:

- Srebrodolskite ($Ca_2Fe_2O_5$): A calcium ferrite phase characteristic of this by-product.
- Magnetite (Fe_3O_4) / Wustite (FeO): Iron oxides contributing to the high density of the material.
- Akermanita ($Ca_2MgSi_2O_7$): A sorosilicate mineral.
- Quartz (SiO_2): Present as a minor crystalline phase.
- Portlandite ($Ca(OH)_2$) / Calcite ($CaCO_3$): Detected due to partial surface hydration and carbonation of free CaO upon storage.

S1.3.- Microstructure and morphology (SEM)

The surface morphology of the slag particles was analyzed using Scanning Electron Microscopy (SEM). Representative micrographs of the material are available in Figure 4a of Matthaiou et al. (2019).

As detailed in the referenced microscopy analysis, the slag particles exhibit a highly irregular geometry characterized by rough textures and sharp edges. This specific morphology is critical for the mechanochemical mechanism proposed in the current study. The abrasive nature of these non-spherical particles enhances the physical disruption of the sludge flocs (Extracellular Polymeric Substances - EPS) during agitation, facilitating the penetration of alkalinity into the floc core and helping to overcome the "calcium shielding effect" described in the manuscript.

S1.4.- Physical properties

- Density: The material possesses a high density of approximately 3.7 g/cm^3, as reported in Matthaiou et al. (2019). This property ensures effective collision momentum during the mixing process with the sludge.
- Particle Size: For the optimization experiments in this study, the material was milled and sieved to a specific particle size range of 0.6–1 mm. This range was selected to balance the surface area available for chemical dissolution with the mass required for effective mechanical abrasion.

References

[1] Matthaiou, V., Oulego, P., Frontistis, Z., Collado, S., Hela, D., Konstantinou, I. K., Diaz, M., & Mantzavinos, D. (2019). Valorization of steel slag towards a Fenton-like catalyst for the degradation of paraben by activated persulfate. *Chemical Engineering Journal*, 360, 728-739.

[2] González-Tolivia, E., Collado, S., Oulego, P., & Díaz, M. (2022). BOF slag as a new alkalizing agent for the stabilization of sewage sludge. *Waste Management*, 153, 335-346.

S2.- pH evolution of the different alkalising agents

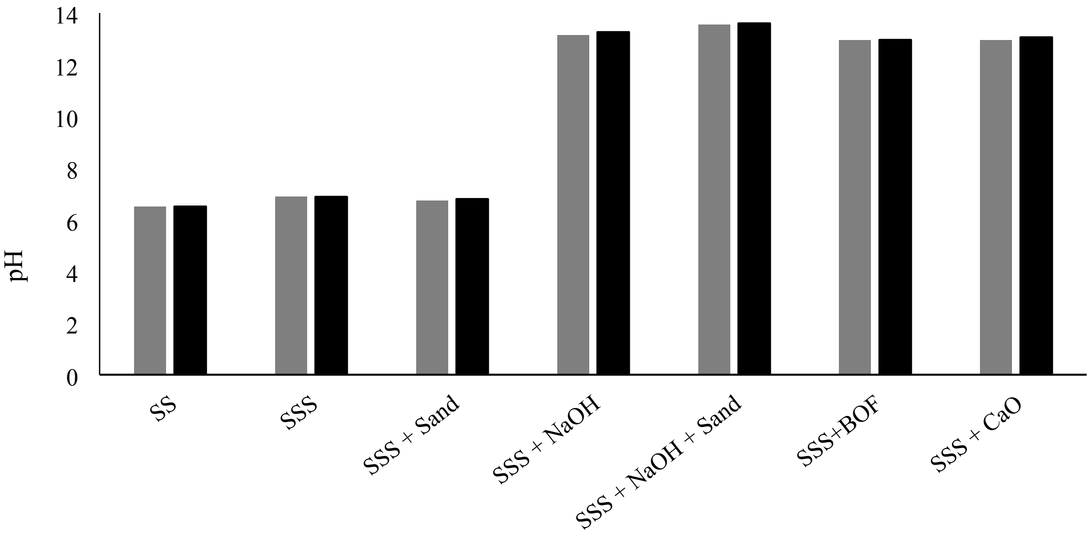

Figure S1. pH at 1 (a grey colour on the graph) and 2 (a black colour) hours of alkaline hydrolysis. Sewage Sludge (SS), Stirred Sewage Sludge (SSS). In all cases: room temperature, 500 mL sludge and 250 rpm.

S3.- Soluble concentration of C, N and P after different treatments of alkaline hydrolysis.

Table S1. Soluble soncentration of C, N and P after different treatments of alkaline hydrolysis. Sewage Sludge (SS), Stirred Sewage Sludge (SSS). In all cases: room temperature, 500 mL sludge and 250 rpm.

	**C** (mol C/gTSS$_0$)	**N** (mol N/gTSS$_0$)	**P** (mol P/gTSS$_0$)
**SS**	3,9E-04	1,1E-03	1,2E-05
**SSS**	6,2E-04	1,8E-04	2,1E-05
**SSS+ Sand**	8,3E-04	5,1E-03	4,2E-04
**SSS+NaOH**	1,9E-02	5,4E-03	3,9E-04
**SSS+ NaOH + Sand**	2,1E-02	5,2E-03	7,2E-05
**SSS+BOF**	6,9E-03	4,1E-03	4,0E-05
**SSS+CaO**	5,5E-03	4,8E-03	2,0E-05
